# One Health in Practice: Using Integrated Bite Case Management to Increase Detection of Rabid Animals in Tanzania

**DOI:** 10.3389/fpubh.2020.00013

**Published:** 2020-02-14

**Authors:** Kennedy Lushasi, Rachel Steenson, Jubilate Bernard, Joel Jackson Changalucha, Nicodem James Govella, Daniel T. Haydon, Husna Hoffu, Felix Lankester, Frank Magoti, Emmanuel Abraham Mpolya, Zacharia Mtema, Hesron Nonga, Katie Hampson

**Affiliations:** ^1^Environmental Health and Ecological Sciences Department, Ifakara Health Institute, Ifakara, Tanzania; ^2^Boyd Orr Centre for Population and Ecosystem Health, College of Medical, Institute of Biodiversity, Animal Health and Comparative Medicine, Veterinary and Life Sciences, University of Glasgow, Glasgow, United Kingdom; ^3^Global Health and Biomedical Sciences, School of Life Sciences and Bioengineering, Nelson Mandela African Institution of Science and Technology, Arusha, Tanzania; ^4^Department of Epidemiology, Ministry of Health, Community Development, Gender, Elderly and Children (MoHCDGEC), Dodoma, Tanzania; ^5^Paul G. Allen School for Global Animal Health, Washington State University, Washington, DC, United States; ^6^Global Animal Health Tanzania, Arusha, Tanzania; ^7^Director of Veterinary Services, Ministry of Livestock Development and Fisheries, Dodoma, Tanzania

**Keywords:** case detection, domestic dog, dog-mediated rabies, elimination, patient management, post-exposure prophylaxis, surveillance, zoonosis

## Abstract

Rabies is a neglected zoonotic disease that causes an estimated 59,000 human deaths worldwide annually, mostly in Africa and Asia. A target of zero human deaths from dog-mediated rabies has been set for 2030, and large-scale control programs are now advocated. However, in most low-income endemic countries surveillance to guide rabies control is weak and few cases of rabies are recorded. There is an urgent need to enhance surveillance to improve timely case detection and inform rabies control and prevention, by operationalizing a “One Health” approach. Here we present data from a study piloting Integrated Bite Case Management (IBCM) to support intersectoral collaboration between health and veterinary workers in Tanzania. We trained government staff to implement IBCM, comprising risk assessments of bite patients by health workers, investigations by livestock field officers to diagnose rabid animals, and use of a mobile phone application to support integration. IBCM was introduced across 20 districts in four regions of Tanzania and results reported after 1 year of implementation. Numbers of bite patient presentations to health facilities varied across regions, but following the introduction of IBCM reporting of bite patients at high-risk for rabies more than doubled in all regions. Over 800 high-risk investigations were carried out, with 49% assessed as probable dog rabies cases on the basis of clinical signs, animal outcome, and rapid diagnostic testing. The status of a further 20% of biting animals could not be determined but rabies could not be ruled out. Livestock field officers reported that use of rapid diagnostic tests (RDTs) were useful for confirming rabies occurrence. Overall, our study provides further evidence that IBCM is a practical approach that can improve rabies detection in endemic countries, and be used to monitor the impact of mass dog vaccinations, including potential to verify rabies freedom. However, the main challenges to implementation are limited training of health workers in rabies, perceived burden of real-time recording and limited resources for livestock field officers to undertake investigations. Nonetheless, IBCM dramatically improved case detection and communication between sectors and we recommend further implementation research to establish best practice and applicability to other settings.

## Introduction

Rabies is a zoonotic disease caused by a virus transmitted through the bite of an infectious animal (1). Around 59,000 people die of rabies each year, with over 99% of these cases occurring in Low- and Middle-Income Countries (LMICs) (2). Yet the disease is entirely preventable through vaccination of dogs to eliminate infection in the reservoir population and by prompt administration of post-exposure prophylaxis (PEP) to people exposed to the virus (1, 3). Control of rabies requires collaboration between public health and veterinary sectors (a “One Health” approach) to manage risks in humans and interrupt transmission in dogs (4). An example of One Health is the Tripartite [World Health Organization (WHO), Food and Agricultural Organization of the United Nations (FAO), and World Organization for Animal Health (OIE)]. These international organizations have united to confront the problem of rabies (5). Nonetheless, the practical coordination of One Health activities by frontline public health and animal health workers remains challenging and this is exemplified by the implementation of rabies surveillance.

Surveillance is essential to control and ultimately eliminate infectious diseases (6). Effective disease surveillance involves the systematic collection and analysis of disease data and timely dissemination of results to guide planning and implementation of control strategies (7–9). Routine analysis of surveillance data can identify changes in disease incidence, including disease outbreaks and should inform public health professionals so as to improve the implementation of interventions and evaluate their impact (10). For rabies, surveillance could include data on persons bitten by rabid animals that are seeking PEP as well as human rabies deaths and on diagnosed animal rabies cases; data that needs to be shared between sectors to inform control measures like dog vaccination campaigns and prevention through provisioning of PEP. Yet, in many rabies endemic countries there are no formal systems used for reporting bite patients, and even if bite patients are reported, information on the risk of rabies is not reported (11). Moreover, limited operationalization of One Health means that the veterinary or public health sectors rarely ever receive information from the other sector to guide their control and prevention activities.

In LMICs where rabies is endemic, there is an urgent need to strengthen health systems and develop effective surveillance tools and response systems (8). Surveillance capacity in both the animal and human health sectors is limited and disease detection is hampered by inadequate laboratory facilities and difficulties in submitting samples to laboratories from rural areas (7, 12, 13). These limitations also render national epidemiological data unreliable with substantial underreporting of both human and animal rabies cases (14) and underestimation of the mortality burden of rabies and its economic impact (15, 16). This leads to rabies control not being prioritized by decision makers against other competing public health concerns.

Integrated Bite Case Management (IBCM) is an approach for rabies surveillance that directly and formally links workers in public health and veterinary sectors to assess the risk of rabies among animal bite patients and biting animals, respectively (17). IBCM has been promoted to increase rabies case detection (17), improve the administration and cost-effectiveness of PEP (18), and as a potential surveillance strategy for verifying freedom from rabies (19). The objective of this study was to determine whether IBCM could be implemented in Tanzania and what potential impact this could have. Here we present results from piloting IBCM in four regions of Tanzania, describing the challenges to implementation and the perceived benefits.

## Methodology

### Study Area

**Figure 1:**
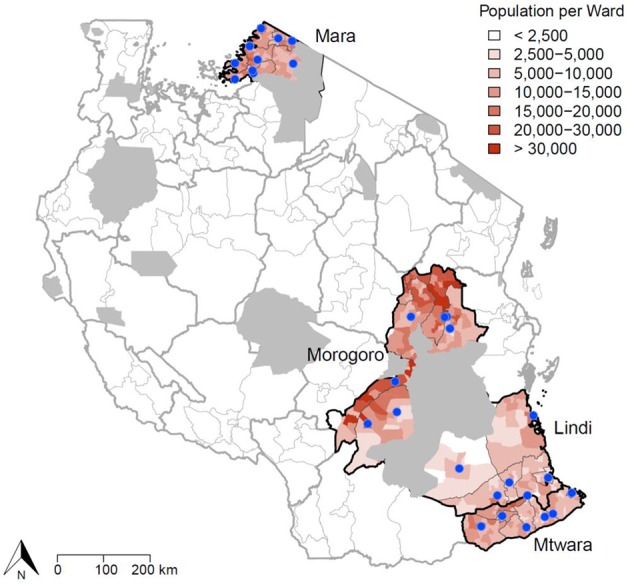
Study area in Tanzania where IBCM was introduced. The blue dots indicate health facilities/hospitals in the districts implementing IBCM where PEP is provided (*n* = 35). The population density of each ward is also illustrated, with wildlife protected areas shown in gray. Human activities are prohibited in wildlife protected areas and these areas are uninhabited.

The study was undertaken across 20 districts in 4 regions in Southern, Central, and Northern Tanzania. IBCM was introduced into Mtwara and Mara regions in June 2018, Lindi region in July 2018 and into Morogoro region in August 2018 (Figure 1). The total human population within these regions was estimated at 7,100,000 in 2019, projected from the 2012 Population and Housing census survey (20). The average human: dog ratio (HDR) in these settings was estimated at 30:1 (21), but varied across districts, giving a dog population in 2019 of around 250,000. The study areas comprise a range of cultural settings with mainly agro-pastoralists in Mara region, agro-pastoralists and farmers in Morogoro region, while farming and fishing dominate in Southern Tanzania.

### IBCM Framework in Tanzania

**Figure 2:**
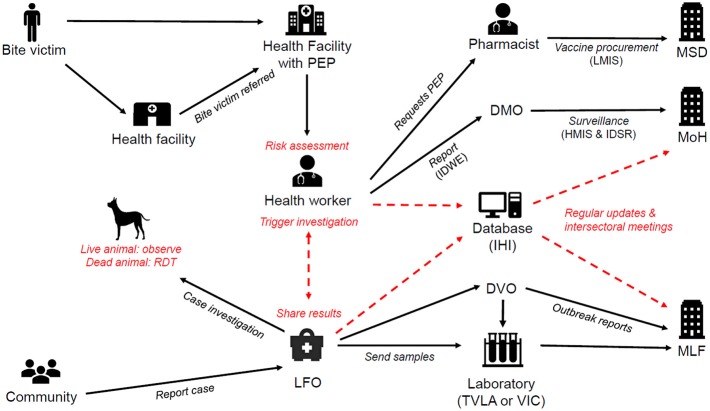
IBCM framework in Tanzania. Red text and arrows indicate interventions introduced as part of IBCM. The existing health systems and reporting structures under the Ministry of Health (MoH) and Ministry of Livestock and Fisheries (MLF) are shown in black and include: the Medical Stores Department (MSD), District Medical Offices (DMO), District Veterinary Offices (DVO), Veterinary Investigation Centres (VIC), the Tanzania Veterinary Laboratories Agency (TVLA), Livestock Field Officers (LFO), the Integrated Disease Weekly Ending (IDWE) surveillance and reporting system, the Logistic Management Information System (LMIS), the Integrated Disease Surveillance and Response system (IDSR) and the Health Management Information System (HMIS). Ifakara Health Institute (IHI) hosts the database server for the IBCM. RDTs are Rapid Diagnostic Tests.

Figure 2 illustrates how an IBCM approach was developed for integration within the existing health and veterinary sectors in Tanzania. The introduction of IBCM involved training health workers to undertake risk assessments and Livestock Field Officers (LFOs), a paraprofessional cadre working within the Ministry of Livestock and Fisheries, to undertake animal investigations. IBCM involves undertaking risk assessments for all patients who present to health facilities with animal bites to determine whether they were bitten by potentially rabid animals or normal healthy animals and to ensure that PEP is correctly administered to exposed individuals to prevent the onset of rabies. Epidemiological investigations should be conducted for animals that bit people to diagnose animal rabies cases. Through these investigations, other exposed individuals may be identified and referred to health facilities that offer PEP.

### The IBCM Application

A mobile phone-based surveillance system for rabies previously developed and set up in southern Tanzania (22) was adapted as the basis for an IBCM application (app) for android phones with a web-based interface (dashboard). The app included risk assessment forms for completion by health workers (Appendix 1) and epidemiological investigation forms for LFOs that also cover sample collection (Appendix 2). The forms use mainly multichoice selections to minimize free-text data entry. The dashboard was developed to monitor submitted records and is accessible via the app or a password protected website. The app was developed using Waterfall development methodology starting with requirement solicitation followed by design, testing, deployment, and maintenance (23). The app is hosted on Google Playstore and has been updated to fix bugs and add new features as required. Data can be accessed via the dashboard by government stakeholders including regional and district veterinary and health officers, who provide feedback to their respective health workers and LFOs on the data being collected.

In February and March 2019, additional functionality was added to the app so that “high-risk” bites were identified following risk assessments by health workers. In response to a high-risk bite being identified, an automated alert would be sent to designated LFOs to trigger an investigation. A “high-risk” investigation was triggered if one of the following criteria were met: (i) a person was bitten by an animal that displayed at least one sign suggestive of rabies (e.g., excessive salivation, paralysis—see Appendix 1 for indicative signs), (ii) a person was bitten by an animal that subsequently disappeared or died or was of unknown origin, (iii) a person was bitten by a wild animal, (iv) a person presented to a health facility with symptoms of rabies. If one of these conditions were met, an automated alert would be triggered when the health worker submitted the risk assessment form. The generated message for the LFO contained the patient ID, their name and location details, including their village and phone number to facilitate the investigation. Automated alerts to LFOs were only generated on a bite patients first visit to a health facility and not for their subsequent visits. Bite patients were given a vaccination card on receipt of their 1st PEP dose and were required to present the card on subsequent visits. The card contained the patient name, age, village and district, date bitten, and PEP dates following the newly recommended 1-week ID regimen (24). If a bite victim sought care but PEP was unavailable, a patient ID was still generated and an alert sent to trigger the investigation. The victim was advised to travel to another health facility for PEP and was given a vaccination card containing their ID and other details indicating that they required PEP. This enabled the next facility to provide PEP to the victim without triggering another investigation.

### Training of Government Personnel

At least one government health facility offers PEP in each district, with a few districts having more than one government facility providing PEP (Figure 1). In each district in the study area, two health workers from each government facility that offers PEP and one LFO were chosen and trained to be focal rabies personnel. The trained health workers were from the immunization departments of each district hospital. To ensure all bite victims who presented to the hospital for treatment were captured, all health and medical attendants working at the Out Patient Department (OPD) were also informed by the respective hospital authorities to refer bite victims to the immunization departments. A joint on-job training was held with LFOs brought to health facilities in their districts and together with health workers they were trained in IBCM. Specifically health workers were trained to undertake risk assessments of bite patients, while the LFOs were trained on how to conduct epidemiological investigations. To maintain implementation of IBCM monthly phone credit was provided to all focal persons (1 GB per month) and reimbursement or advance payment to LFOs for fuel to undertake investigations (typically 10,000–20,000 Tsh per investigation).

The protocol for LFOs involved first conducting a phone consultation with either the animal bite victim or a relative of the victim whose phone number was recorded during the health worker's risk assessment. In scenarios where the biting animal's information could not be obtained through phone consultation, LFOs were advised to visit the household of the animal owner. If multiple people were attacked by the same animal the LFO was required to record their names, patient ID (if the person had sought care), village, and PEP status on the investigation form. If the biting animal was vaccinated against rabies and did not appear sick, no further investigation was undertaken, however the owner was instructed to observe the animal for 10 days following the bite incident and immediately inform the LFO if any health or behavioral changes were observed. If the investigation revealed the animal was suspected to be rabid, the LFO was advised to check within the community to determine whether any other persons or animals had been exposed. LFOs were trained to collect samples from animals that had been killed or died, and were provided with BioNote rapid diagnostic test (RDT) to test for rabies where possible (25). Following investigations, LFO were advised to inform the health worker of the investigation result. Consent was not sought from patients for undertaking risk assessments or for animal investigations, as both activities are considered part of government duties. However, patients were informed that their data was being recorded electronically to inform an investigation of the biting animal.

Six months after IBCM was first introduced between June and August 2018, a proficiency questionnaire was administered to health workers to assess their knowledge of the clinical signs of rabies and whether they could distinguish rabid from healthy biting animals whilst attending animal bite victims. This was done in February 2019 in Lindi and Mtwara regions, in March 2019 in Morogoro region and in April 2019 in Mara region, and was immediately followed by refresher training and a post-training assessment examining two rabies risk scenarios.

To quantify baseline incidence of bite patient presentations, prior to the introduction of IBCM, we collected paper records from health facilities in the study from the 1st of January 2018. To determine the impact of introducing IBCM, we analyzed records from the IBCM database up until the 1st of August 2019, providing 1 year of data following the introduction of IBCM. We used a chi-squared test to investigate differences in risk classifications pre- and post- implementation of IBCM.

## Results

### Bite Patient Risk Assessments

**Figure 3:**
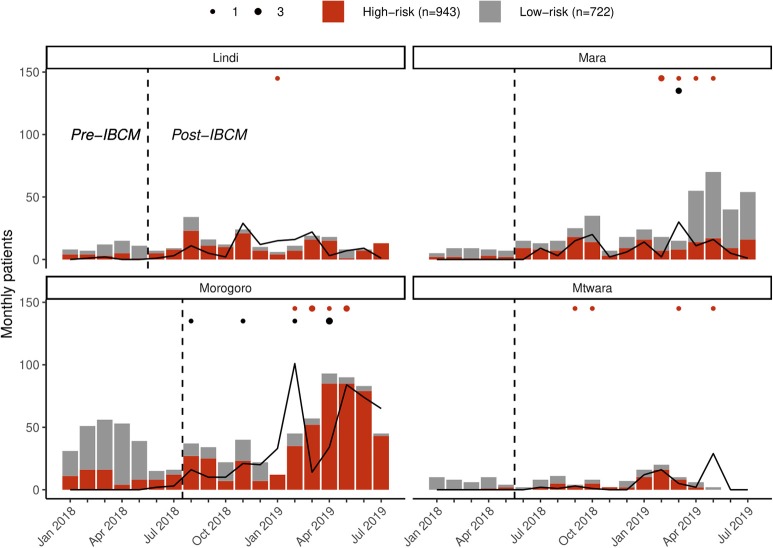
Regional reporting of bites assessed as high risk (red) vs. low risk (gray) and investigations of biting animals (lines) in the study regions. The dotted line indicates when IBCM was implemented in each region; red dots indicate the number of human deaths (*n* = 16) attributable to rabies; black dots indicate the number of positive animal rabies cases (*n* = 8) confirmed through rapid diagnostic tests.

Prior to the introduction of IBCM, an average of 55.7 (range: 15–86) new bite patients presented per month in these regions, with only 26.9% indicating a risk of rabies by the health worker who completed the record (Figure 3). Following the introduction of IBCM, an average of 92.2 (range: 15–174) bite incidents were reported per month, with 64.9% assessed by health workers to be by suspect rabid animals. Overall bite patient presentations corresponded to an incidence of 17.4 bites per 100,000 persons per annum over the study period from January 2018 until August 2019 (from 1 to 64.9 among districts), but a risk of 12.0 rabies exposures /100,0000/year (from 1 to 62.2 among districts) under IBCM (from June 2018 until August 2019), assuming that the health workers risk assessments provide a more accurate indicator of rabies than routine records of bite patients (vs. 4.1/100,0000/year pre-IBCM from January 2018 until June 2018) before the introduction of IBCM (Table 1).

**Table 1:** Patient presentations in study regions before and after the introduction of IBCM.

**Region**	**Pre-IBCM**		**Post-IBCM**	
	**Patient presentation per 100,000 persons per annum**	**% high-risk**	**Patient presentation per 100,000 persons per annum**	**% high-risk**
Lindi	15.0	31.5	19.4	76.0^**^
Mara	5.2	26.3	20.1	39.1
Morogoro	28.1	28.8	22.6	82.9^**^
Mtwara	7.2	7.9	6.7	59.0^**^

Significant differences in the proportions of high-risk patients pre- and post-IBCM are indicated by * < 0.05 and

** *< 0.001, as detected by a chi-squared test*.

Of the bite victims that presented to health facilities following the introduction of IBCM (between July 2018 and August 2019; *n* = 1,291), most were due to bites from domestic dogs (93.0%) with only a few being bitten by wild animals (Lindi, *n* = 3; Morogoro, *n* = 1, Mtwara, *n* = 14, and Mara, *n* = 7). Most bite patients were recorded with scratches or minor wounds (78.0%, *n* = 1,007), while 19.5% (*n* = 252) had more severe wounds and 1.2% (*n* = 16) required hospitalization due to broken bones or infection. One child (aged 2) died as a result of bite injuries. Throughout the study regions, PEP was unavailable for 74 bite patients (5.7%) upon presentation to a health facility, during the period of IBCM implementation. Only 63 of these bite patients were referred to other facilities for PEP with 43 assessed as being suspect rabies exposures. Sixteen human deaths due to rabies were reported within the IBCM study districts between July 2018 and August 2019 (Figure 3) from: Kilwa (1) in Lindi region; Bunda (4) and Serengeti (1) in Mara region; Morogoro Urban (3) and Ulanga (3) in Morogoro region; and Mtwara Rural (1) and Newala (3) in Mtwara region. These deaths were also confirmed through the investigations done by LFOs after the health worker's alert.

### Animal Investigations

**Figure 4:**
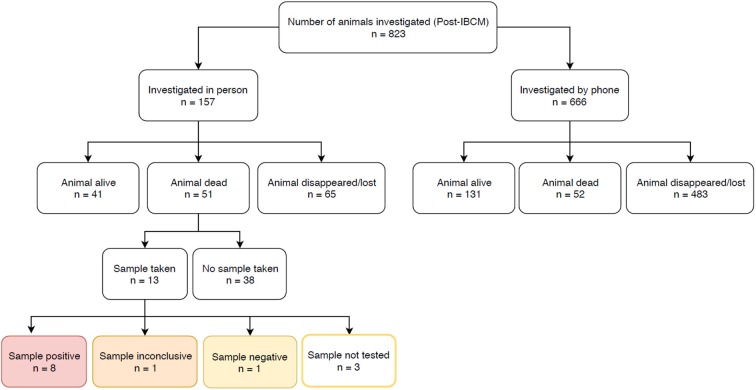
Number of investigations carried out by LFOs between August 2018 and May 2019. The outcomes of rapid diagnostic tests are highlighted.

Prior to the introduction of IBCM, investigations were not carried out as standard by LFOs but were only carried out on an *ad hoc* basis. However, since IBCM began in the study area, 823 investigations have been conducted by LFOs. Seven hundred and seventy-seven investigations were conducted following an alert of a potentially high-risk bite while 46 investigations were carried out following community reports of sick, dead, or biting animals (Figure 3). The number of investigations undertaken following the introduction of IBCM differed between regions, with LFOs investigating an average of 10.3 cases/month in Mara, 9.7/month in Lindi, 40.2/month in Morogoro, and 7.9/month in Mtwara (Figure 3). An outbreak of rabies that began in February 2019 resulted in a surge of investigations in Morogoro region (Figure 3). Out of all the investigations, 157 were carried out in person, and 666 were completed via a phone consultation. From the 157 in-person investigations, 13 samples (8.3%) were collected between August 2018 and August 2019 (Figure 4) and 10 of these were tested with a RDT.

From the investigations, 49.1% (404/823) of biting animals showed at least one clinical sign consistent with rabies and/or were positive following a RDT (*n* = 8/10; one specimen tested negative and one test was inconclusive), while 21.5% (177/823) were determined to be healthy and 4.9% (40/823) to be sick from other causes that were not rabies. The remaining 24.5% (202/823) were classified as unknown status due to insufficient evidence. 20.9% (172/823) of the animals investigated were alive at the time of investigation, allowing for observation of clinical signs; the remaining 79.1% (651/823) had either disappeared (66.6%; 548/823) or were already dead (12.5%; 103/823) at the time of investigation, with a large proportion killed by community members (*n* = 62) or their owner (*n* = 11). Almost all domestic dogs are owned in rural Tanzania, but also almost all domestic dogs roam freely. Therefore, investigations were difficult to resolve if the owner of the biting dog was not known and the dog disappeared following the bite, but such circumstances were assumed to be high-risk and potentially indicative of rabies.

### Veterinary and Health Data Combined (One Health)

**Figure 5:**
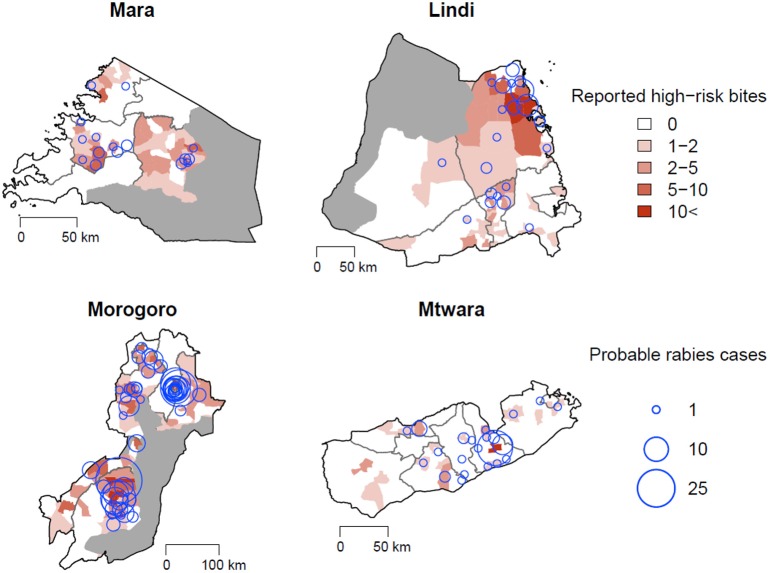
High-risk bites per ward reported through health facilities (red polygons) and probable cases confirmed through LFO investigations (blue circles). Protected areas are overlaid in gray.

The high-risk bites and animals assessed as suspect for rabies were generally widespread across the study regions (Figure 5). Both health workers and LFOs reported similar criteria about biting animals that they assessed to be suspect rabid. Health workers considered suspect rabid animals to show unprovoked aggression (including attempting to bite and grip people, animals, or objects without feeding; 45.2%), excessive salivation (10.4%), restlessness (6.4%), and/or abnormal vocalization (6.9%). In 23.1% (298/1,291) of bite patients, the health worker did not report any clinical signs for the biting animal, yet still classified 117 them as suspect rabies, apparently because the animal was unknown or the attack unprovoked. On investigation of high-risk bites, LFOs also reported animals displaying unprovoked aggression (49.5%), abnormal vocalization (16.8%), restlessness (9.8%), and/or excessive salivation (8.7%). LFOs did not report any clinical signs in 14.2% (117/823) of investigations, but considered 36 of these animals to be suspect rabid on the basis of other unreported information.

### Assessment of Health Worker Knowledge

The proficiency testing indicated that all health workers could identify at least three clinical signs in animals consistent with rabies. Excessive salivation (94%), restlessness (86%), unprovoked aggression (86%), and abnormal vocalization (74%) were the most commonly identified signs, but a few respondents also identified paralysis (34%), and diurnal activity amongst nocturnal wildlife (24%) as well as a lack of fear among wildlife (16%) as clinical signs. However, only 66% of respondents considered a bite from an unknown animal as suspicious for rabies.

During proficiency testing most health workers stated that the wound severity would affect their recommendation for PEP and whether they would inform LFOs to investigate. Health workers reported that they were most likely to classify an exposure as high-risk and recommend PEP when treating severe wounds (wounds requiring hospitalization 90%, large wounds 96%, minor wounds 86%, and scratches 62%) and were also more likely to request LFOs to investigate severe bites (fatal wounds 78%, wounds requiring hospitalization 90%, large wounds 88%, minor wounds 70%, and scratches 56%). Only 78% of health workers indicated they would inform an LFO if they received a patient presenting with clinical signs of rabies. Following the refresher training, 74% of health workers were able to correctly recommend PEP to a patient bitten by an unknown suspected dog who had been delayed in seeking treatment.

## Discussion

Implementing IBCM demonstrated important public health impacts of rabies in Tanzania and the need to improve PEP access to prevent human rabies deaths as well as mass dog vaccination to control the disease at source. Reports of bites by suspected rabid dogs more than doubled under IBCM, and a large proportion of biting animals were identified as probable rabies cases upon investigation. Over half of patients presenting to clinics were assessed to have been bitten by suspect rabid dogs and therefore urgently required PEP. But, shortages of PEP occurred and human rabies deaths were reported from every region. Although it was possible to implement IBCM across this large geographic area, some activities were challenging, including recognition of indicative signs of rabies by health workers and investigations leading to sample collection by LFOs. Extended training could go some way to addressing these difficulties but limited resources are a constraint. Nonetheless, IBCM shows considerable promise for improving case detection and communication between sectors, and further implementation research is warranted.

IBCM showed promise as a tool to support rabies surveillance. Specifically, IBCM increased case detection, and generated data from health facilities that is much more useful for assessing the impact of PEP than numbers of bite patients alone, which may often not reflect rabies incidence directly (11, 26–28). The use of the mobile phone application was generally successful and both health workers and LFOs were enthusiastic about how IBCM improved intersectoral collaboration and understanding of the rabies problem, with LFOs particularly positive about using RDTs to confirm rabies. This was most evident during the response to a rabies outbreak in the first half of 2019 in Morogoro region, where several deaths occurred and dog cases were confirmed. The incidence of bite patients, suspect rabies exposures and deaths identified through IBCM in Morogoro was similar to numbers reported from the investigation of a previous outbreak in the region in 2007 (29), whereas incidence in the other districts was relatively low, likely because of previous dog vaccination campaigns.

Limitations of our study restricted the conclusions we were able to draw. For example, we introduced IBCM to the government designated hospital in each district that offers PEP, but private referral hospitals, such as St Francis in Kilombero District or Maneromango in Nachingwea district, were not included in the study, though they also offer PEP. Bite victims who directly attended these facilities (sometimes due to PEP stockouts elsewhere) were therefore not captured by IBCM. In addition, bite victims who never attended any of the health facilities and developed rabies and died at home were not captured by either IBCM or routine surveillance, leading to underestimation of the disease burden. Integration of private facilities may be needed in future if Tanzania is to bring rabies under control and IBCM is used to verify rabies freedom. Training was given to government workers and follow up provided by the research team, with one assistant remotely supporting all four regions. Without such technical support it may be difficult for the government to scale up IBCM to other parts of the country. More generally, district councils may vary in terms of follow up, levels of staff training and availability of funds that affects the quality of operations. These points likely apply to other LMIC settings and should be considered if efforts are made to improve PEP access and introduce IBCM (30). Nonetheless, once trained, both health workers and LFOs were able to fully implement IBCM independent of the research team, and IBCM activities were mostly adopted and integrated within routine duties. Further work will be required to fully understand sustainability of IBCM.

Trained practitioners are indispensable to an effective health system and this applies directly to IBCM. Two or three health workers were trained to implement IBCM in each facility. This increased the workload of these health workers and they felt deserved extra payment. Some health workers were also re-assigned to other facilities or departments, which required the recruitment of replacements who were trained remotely via phone. The level of knowledge and familiarity with smartphones also differed between users (both health workers and LFOs), and for a few using an app was challenging. In some facilities in Southern Tanzania only very few bite victims presented, and as a result health workers in these areas (7 out of 63 across the study) needed reminding to conduct risk assessments, and were encouraged to use the IBCM guide provided during their training to recall procedures. Generally, health workers receive only limited professional training in rabies in Tanzania. The proficiency training we provided aimed to boost their ability to recognize signs of rabies, because of the difficulty that health workers showed in fully understanding rabies risks and indicative clinical signs in animals. Providing regular incentives such as training outside their workplace or in monetary terms could potentially help improve their performance, but is a challenge for sustainability. Government supported training to reinforce IBCM, particularly over time and with staff turnover, could benefit sustainability. But it is likely that an ongoing support person would be required for troubleshooting, ideally a designated government employee.

From the animal health side, obtaining samples for diagnosis was difficult. Timely investigations are critical for confirming cases as well as for detecting other exposures, in both animals and people. Delays compromise sample collection opportunities and heighten risks for those who have not sought care. However, many cases that require investigation are far from district headquarters and the focal LFO responsible for sample collection. It is difficult for LFOs with limited resources and inadequate transport to reach these cases. Nevertheless, our experience suggests that sample collection could improve, with emphasis on timely submission of risk assessments by health workers, additional training of LFOs based in more remote areas. Unfortunately at the start of the study we were unable to fully equip LFOs with RDTs and so not all samples were collected and tested, but feedback from LFOs suggested that the ability to test samples was also a strong incentive for collection. One Health is widely promoted (31) and is highly recommended for rabies control and prevention (12). IBCM represents a formal means of practicing intersectoral collaboration. We suggest that further joint discussions about surveillance findings amongst practitioners, including engagement with the regional and council health management teams, could help reinforce IBCM and ultimately promote better implementation of One Health and rabies control and prevention activities. Surveillance investments typically focus on laboratory diagnostics and infrastructure, but resourcing health workers to conduct risk assessments and LFOs to carry out investigations would be a critical first to improve rabies case detection.

Vaccination of all persons exposed to a suspected rabid animal is an effective approach to protect people from rabies (5, 32). However, rabies vaccines in Tanzania are in short supply, so unnecessary use can also limit availability for those most in need (33–35). While risk assessments indicate some potential for more judicious use of PEP in patients bitten by clearly healthy animals, the number and proportion of those presenting due to healthy animal bites is small compared to some settings, particularly in Asia and the Americas (27, 36). Risk assessments to determine PEP decisions needs to be both sensitive and specific. PEP should always be recommended if there is any doubt concerning the risk of rabies, and therefore risk assessments with low sensitivity could lead to human rabies cases if PEP is either not initiated or delayed in a genuine rabies exposure, whilst risk assessments with low specificity could lead to people receiving PEP unnecessarily, incurring expenses and potentially limiting supply for those in need. A challenge for judicious PEP administration is that bite victims may demand PEP for bites from healthy animals, particularly in areas with recent rabies cases, and will cover any costs required or demand PEP and associated costs be covered by dog owners. Our findings suggests that there is quite limited scope for more prudent PEP use in Tanzania, and that increasing PEP access should be the first priority. Nonetheless, the use of IBCM in such highly endemic settings could sensitize practitioners to the risks of rabies, and given limited diagnostic capacity and PEP availability, may be useful to guide PEP recommendations and prevent unnecessary overuse, particularly with a view to progressing toward elimination (18, 19, 33, 37).

## Conclusion

In Tanzania, animal disease surveillance falls under the Ministry of Livestock and Fisheries, while the Ministry of Health deals with bite victims. An intersectoral programme, such as the One Health Coordination Unit, under the Prime Ministers' office encourages both sectors to work together in a practical way, but coordinating rabies prevention and control between the two sectors has always been a challenge. IBCM has helped to integrate these sectors and generates more accurate surveillance data that can guide policy decisions and public health measures. IBCM improved intersectoral communication, helped to identify rabies-exposed bite victims requiring PEP, facilitated follow up of cases, and encouraged LFOs to rapidly test cases during investigations. Surveillance is crucial to guiding effective patient management decisions, disease control interventions and for verifying disease elimination. A well-established surveillance system will be essential to evaluate the impact of mass dog vaccination programmes and to ensure rapid responses to outbreaks. IBCM appears to be a practical and promising approach to improve case detection and was extremely useful during the outbreak of rabies in Morogoro region. Whether IBCM can confirm the interruption of disease transmission will depend on implementation. Until now, many practices for rabies control and prevention are still weak in LMICs with endemic rabies (9), and will need strengthening to achieve the zero by 30 goal. We encourage greater use of IBCM and recommend further implementation research to develop best practice for IBCM in different settings, and evaluate its potential to support the goal of elimination of dog-mediated rabies.

## Data Availability Statement

Anonymized data and code to reproduce the results, including the figures and tables are available on the GitHub repository: https://github.com/LushasiK/IBCM_TZ_Frontiers.

## Ethics Statement

The study was approved by the Medical Research Coordinating Committee of the National Institute for Medical Research of Tanzania, with approval number NIMR/HQ/R.8a/vol.IX/2788, the Ministry of Regional Administration and Local government with the reference number AB.81/288/01, and the Institutional ethical review board of Ifakara Health Institute with approval number IHI/IRB/No: 22-2014.

## Author Contributions

KL and KH conceived the study. KL, ZM, and KH designed the study. KL, HH, FM, ZM, and JC collected the data. KL, RS, and KH analyzed the data. KL reviewed the literature and wrote a first draft. RS, KH, and FL redrafted the manuscript. EM, HN, DH, JB, and NG critically reviewed and approved the final manuscript for submission.

### Conflict of Interest

The authors declare that the research was conducted in the absence of any commercial or financial relationships that could be construed as a potential conflict of interest.

## Supplementary Material

Appendix 1 Risk assessment form for Health Workers.

Appendix 2 Investigation form for Livestock Field Officers.

## References

[1] Jackson AC. Current and future approaches to the therapy of human rabies. Antiviral Res. (2013) 99:61–7. 10.1016/j.antiviral.2013.01.003 23369672

[2] Hampson K Coudeville L Lembo T Sambo M Kieffer A Attlan M Estimating the global burden of endemic canine rabies. PLoS Negl Trop Dis. (2015) 9:e0003709 10.1371/journal.pntd.0003709 25881058 PMC4400070

[3] Fooks AR Banyard AC Horton DL Johnson N McElhinney LM Jackson AC. Current status of rabies and prospects for elimination. Lancet. (2014) 384:1389–99. 10.1016/S0140-6736(13)62707-5 24828901 PMC7159301

[4] Rushton J Häsler B De Haan N Rushton R. Economic benefits or drivers of a one health approach: why should anyone invest? Onderstepoort J Vet Res. (2012) 79:461. 10.4102/ojvr.v79i2.461 23327381

[5] World Health Organization Rabies vaccines: WHO position paper, April 2018 – Recommendations. Vaccine. (2018) 36:5500–3. 10.1016/j.vaccine.2018.06.061 30107991

[6] Townsend SE Lembo T Cleaveland S Meslin FX Miranda ME Putra AAG. Surveillance guidelines for disease elimination: a case study of canine rabies. Comp Immunol Microbiol Infect Dis. (2013) 36:249–61. 10.1016/j.cimid.2012.10.008 23260376 PMC3693035

[7] Karimuribo ED Sayalel K Beda E Short N Wambura P Mboera LG. Towards one health disease surveillance: the Southern African Centre for infectious disease surveillance approach. Onderstepoort J Vet Res. (2012) 79:454. 10.4102/ojvr.v79i2.454 23327374

[8] Halliday J Daborn C Auty H Mtema Z Lembo T Bronsvoort BM. Bringing together emerging and endemic zoonoses surveillance: shared challenges and a common solution. Philos Trans R Soc B Biol Sci. (2012) 367:2872–80. 10.1098/rstb.2011.0362 22966142 PMC3427560

[9] Tambo E Ai L Zhou X Chen JH Hu W Bergquist R. Surveillance-response systems: the key to elimination of tropical diseases. Infect Dis Poverty. (2014) 3:17. 10.1186/2049-9957-3-17 24971165 PMC4071800

[10] Zhang H Lai S Wang L Zhao D Zhou D Lan Y. Improving the performance of outbreak detection algorithms by classifying the levels of disease incidence. PLoS ONE. (2013) 8:e71803. 10.1371/journal.pone.0071803 23977146 PMC3747136

[11] Wambura G Mwatondo A Muturi M Nasimiyu C Wentworth D Hampson K. Rabies vaccine and immunoglobulin supply and logistics : challenges and opportunities for rabies elimination in Kenya. Vaccine. (2019) 37 (Suppl. 1):A28–34. 10.1016/j.vaccine.2019.05.035 31326251 PMC7612384

[12] Lechenne M Mindekem R Madjadinan S Oussiguéré A Moto DD Naissengar K. The importance of a participatory and integrated one health approach for rabies control: the case of N'Djaména, chad. Trop Med Infect Dis. (2017) 2:43. 10.3390/tropicalmed2030043 30270900 PMC6082095

[13] Mazigo HD. Rabies in Tanzania: the need for a national control programme. Tanzan J Health Res. (2011) 13:87–8. 10.4314/thrb.v13i2.63652 25566604

[14] Broban A Tejiokem MC Tiembré I Druelles S L'Azou M. Bolstering human rabies surveillance in Africa is crucial to eliminating canine-mediated rabies. PLoS Negl Trop Dis. (2018) 12:e0006367. 10.1371/journal.pntd.0006367 30188896 PMC6126826

[15] Nel LH. Discrepancies in data reporting for rabies, Africa. Emerg Infect Dis. (2013) 19:529–33. 10.3201/eid1904.120185 23628197 PMC3647406

[16] Taylor LH Knopf L. Surveillance of human rabies by national authorities - a global survey. Zoonoses Public Health. (2015) 62:543–52. 10.1111/zph.12183 25683444

[17] Wallace RM Reses H Franka R Dilius P Fenelon N Orciari L. Establishment of a canine rabies burden in Haiti through the implementation of a novel surveillance program. PLoS Negl Trop Dis. (2015) 9:e0004245. 10.1371/journal.pntd.0004245 26600437 PMC4657989

[18] Undurraga EA Meltzer MI Tran CH Atkins CY Etheart MD Millien MF. Cost-effectiveness evaluation of a novel integrated bite case management program for the control of human rabies, Haiti 2014-2015. Am J Trop Med Hyg. (2017) 96:1307–17. 10.4269/ajtmh.16-0785 28719253 PMC5462564

[19] Hampson K Abela-ridder B Brunker K Bucheli STM Carvalho M Caldas E Surveillance to establish elimination of transmission and freedom from dog- mediated rabies. bioRxiv. 096883:1–22. 10.1101/096883

[20] Government of the United Republic of Tanzania and Ministry of Finance and Planning Tanzania Human Development Report 2017. (2018). Available online at: http://www.esrf.or.tz/docs/thdr2017launch.pdf (accessed June 2019).

[21] Sambo M Johnson PCD Hotopp K Changalucha J Cleaveland S Kazwala R. Comparing methods of assessing dog rabies vaccination coverage in rural and urban communities in Tanzania. Front Vet Sci. (2017) 4:33. 10.3389/fvets.2017.00033 28352630 PMC5348529

[22] Mtema Z Changalucha J Cleaveland S Elias M Ferguson HM Halliday JEB. Mobile phones as surveillance tools: implementing and evaluating a large-scale intersectoral surveillance system for rabies in Tanzania. PLoS Med. (2016) 13:e1002002. 10.1371/journal.pmed.1002002 27070315 PMC4829224

[23] Sommerville I Software Engineering. 10th edition. Harlow: Addison-Wesley (2010).

[24] Tarantola A Ly S Chan M In S Peng Y Hing C. Intradermal rabies post-exposure prophylaxis can be abridged with no measurable impact on clinical outcome in Cambodia, 2003–2014. Vaccine. (2018) 37(Suppl. 1):A118–27. 10.1016/j.vaccine.2018.10.054 30454946

[25] Lechenne M Naissengar K Lepelletier A Alfaroukh IO Bourhy H Zinsstag J. Validation of a rapid rabies diagnostic tool for field surveillance in developing countries. PLoS Negl Trop Dis. (2016) 10:e0005010. 10.1371/journal.pntd.0005010 27706156 PMC5051951

[26] Rajeev M Edosoa G Hanitriniaina C Fy S Guis H Ramiandrasoa R. Healthcare utilization, provisioning of post-exposure prophylaxis, and estimation of human rabies burden in Madagascar. Vaccine. (2018) 37(Suppl. 1):A35–44. 10.1016/j.vaccine.2018.11.011 30509692 PMC7612383

[27] Rysava K Miranda ME Zapatos R Lapiz S Rances P Roces MC. On the path to rabies elimination: the need for risk assessments to improve administration of post-exposure prophylaxis. Vaccine. (2018) 37(Suppl. 1):A64–72. 10.1016/j.vaccine.2018.11.066 30573356 PMC6863041

[28] Benavides JA Megid J Campos A Rocha S Vigilato M Hampson K. An evaluation of Brazil's surveillance and prophylaxis for canine rabies. PLoS Negl Trop Dis. (2019) 13:e0007564. 10.1371/journal.pntd.0007564 31381564 PMC6709922

[29] Sambo M Cleaveland S Ferguson H Lembo T Simon C Urassa H. The burden of rabies in Tanzania and its impact on local communities. PLoS Negl Trop Dis. (2013) 7:e2510. 10.1371/journal.pntd.0002510 24244767 PMC3820724

[30] Cauchemez S Bourhy H. Improving the provision of rabies post-exposure prophylaxis. Lancet Infect Dis. (2019) 19:12–13. 10.1016/S1473-3099(18)30606-6 30472177

[31] Tiensin T Chuxnum T How Can We Progress the Cooperation Between Animal Health Sector and the Public Health Sector? (2015). Available online at: http://www.oie.int/fileadmin/Home/eng/Publications_%26_Documentation/docs/pdf/TT/2015_ASI2_Tiensin.pdf (accessed August 2019).

[32] Hampson K Dobson A Kaare M Dushoff J Magoto M Sindoya E. Rabies exposures, post-exposure prophylaxis and deaths in a region of endemic canine rabies. PLoS Negl Trop Dis. (2008) 2:e339. 10.1371/journal.pntd.0000339 19030223 PMC2582685

[33] Medley A Millien M Blanton J Ma X Augustin P Crowdis K Retrospective cohort study to assess the risk of rabies in biting dogs, 2013–2015, republic of Haiti. Trop Med Infect Dis. (2017) 2:14 10.3390/tropicalmed2020014 PMC6082081 30270873

[34] Shantavasinkul P Wilde H. Postexposure Prophylaxis for Rabies in Resource-Limited/Poor Countries. 1st ed. Bangkok: Elsevier Inc. (2011). 10.1016/B978-0-12-387040-7.00013-5 21601051

[35] Etheart MD Kligerman M Augustin PD Blanton JD Monroe B Fleurinord L. Effect of counselling on health-care-seeking behaviours and rabies vaccination adherence after dog bites in Haiti, 2014–15: a retrospective follow-up survey. Lancet Glob Heal. (2017) 5:e1017–25. 10.1016/S2214-109X(17)30321-2 28911750 PMC5639700

[36] Valenzuela LM Jayme SI Amparo ACB Taylor LH Dela Cruz MPZ Licuan DA. The ilocos norte communities against rabies exposure elimination project in the philippines: epidemiological and economic aspects. Front Vet Sci. (2017) 4:54 10.3389/fvets.2017.00054 28484703 PMC5402182

[37] Abela-Ridder B Knopf L Martin S Taylor L Torres G De Balogh K. 2016: The beginning of the end of rabies? Lancet Glob Heal. (2016) 4:e780–1. 10.1016/S2214-109X(16)30245-5 27692777

