# Supplementary material for: One Health in Practice: Using Integrated Bite Case Management to Increase Detection of Rabid Animals in Tanzania
Source: Front Public Health. 2020 Feb 14;8:13. doi: 10.3389/fpubh.2020.00013 (PMC7034360; doi:10.3389/fpubh.2020.00013)
Supplement: Appendix 1 — Risk assessment form for Health Workers. [file Table_1.DOC]

**Human investigation form**

1. Name of Health facility: ***__________(entered automatically)***
2. Visit date: ____________ ***(entered automatically)***
3. **Names of the victim:**

3.1 Sir Name (Jina la ukoo):__ 3.2 given Names (2): ___

- 1. Common name known; ___3.4 Age: ______ 3.5 Sex: __

3.6 Can you provide phone number ... Yes/No?

3.7 If yes, Phone No; ___________ ***(restrict to 10 digits)***

3.8 Phone status (Own/neighbour/no phone) *select*

1. Human ID: ***(created automatically by Zac/Frank)***
2. **Location** *(Select from the list)*

Region: __________District:______ Village: ________ Type write Village if not indicated on the list ______

1. **Bite history**

6.1 Bite status visit (If 1^st^ dose complete the rest of questions, if 2^nd^,3rd or 4^th^ ***select that corresponds and skip to qn. 14***; or positive clinical signs): Tick that corresponds

6.2 Date bitten: ___________ 6.3. Date reported to hospital: ____________

- 1. Biting animal: *Select from the list below*

Dogs, cats, livestock (cow, goat/sheep, pig), wildlife (specify), human

1. **Risk assessment (Tick)**
   1. Type of the animal: domestic/ wildlife (Tick)
      1. animal signs (Tick):
      - Unprovoked aggression (incl. attempting to bite and grip people, animals, or objects, without feeding
      - Excessive salivation
      - Unexplained dullness/lethargy
      - Hyper sexuality
      - Paralysis
      - Abnormal vocalization
      - Restlessness
      - Running without reason
      - Tameness/loss of fear of humans (wildlife)
      - Active during day (wildlife)

**None of the above**

- - 1. Feeding puppies (Yes /No), Eating (Yes /No), Normal behaviour i.e. aggressive dog (Yes /No)
    2. is the dog/animal known in the community? (Yes, proceed to 7.1.4 /No)
    3. If yes, name of the dog owner: ___________, Village: ____________
  1. **The victim:** Noise (speaking/shouting) (Yes/No); running (Yes/No); Aggressive (Yes/No); scared of dogs (Yes/No); throw anything at the dog (Yes/No); playing (Yes/No); approaching the dog (Yes/No); NO provocation (Yes/No)
  2. **Environment:** Chained (Yes/No); Fenced (Yes/No); with no owner (Yes/No); with owner (Yes/No); Lots of people (Yes/No), Lots of dogs (Yes/No); on its property (Yes/No),

Dog came out of nowhere: Ticks

1. Is the animal still alive? Yes/No (select)
2. Veterinary department consulted for follow up; (Yes/No): Tick
3. Rabies Assessment decision: ☐ Healthy ☐ suspicious for of rabies ☐ Sick, not rabies ☐ Unknown (Tick)
4. **Bite site:** Tick that applies

☐ Head/neck ☐ Trunk ☐ Arms/Hands ☐ Legs/ Feet

1. **Bite details:** Tick the level that applies

☐ Scratch: ☐ Minor wounds ☐ large wounds ☐ Severe (broken bones)

☐ Severe (hospitalization) ☐ fatal bite (bitten to death).

1. Treatment of victim (*check all that apply*)

☐ Nothing ☐ Tetanus ☐ Wound washing

☐ Antibiotics ☐ Immunoglobulin

1. PEP availability and recommendations
   1. PEP advised & available (If yes, how used? (ID/IM) *select the correct*
   2. PEP advised & referred (If yes, where? *Free text*)
   3. PEP not advised
2. **Comment (if any):**
