# Supplementary material for: One Health in Practice: Using Integrated Bite Case Management to Increase Detection of Rabid Animals in Tanzania
Source: Front Public Health. 2020 Feb 14;8:13. doi: 10.3389/fpubh.2020.00013 (PMC7034360; doi:10.3389/fpubh.2020.00013)
Supplement: Appendix 2 — Investigation form for Livestock Field Officers. [file Table_2.DOC]

Animal investigation form

**Date of investigation: _ _/_ _ /_ _ _ _ (automatically entered)**

**Name of investigation officer: (automatically entered)**

**Animal ID: (**Is investigation linked to bite incident, if Y use ID from bite patient, otherwise create ID automatically): This needs to be automatically created

1. **Location of event**: (*select from the list provided*)

Region__________ District_______ Village _________ other village ________ *(if not in the list hand type the village)*

1. Type of Investigation (Tick): Phone Consultation / In-Person Investigation
2. **Reported from** (mark all that apply)

☐ Health centre/worker

☐ Veterinarian

☐ Community members’ _____________________

☐ other (*please specify*) ________________

1. **Reason for report** *(Tick)*:

Human Exposure (bite/scratch) ☐ Suspect rabid animal ☐ Hit by car ☐ animal found dead

☐ animal is sick ☐ person is sick ☐ other (*specify*)

1. **Type of animal:** Select

Dogs, cats, livestock (cow, goat/sheep, pig), wildlife (specify), human

1. **Was this animal**: ☐ Unowned ☐ Owned ☐ Unknown (TICK)
   1. if owned, names of animal owner______
2. How many people were bitten/scratched by the animal? ___________
3. Dropdown to record name of each person bitten, sought PEP Y/N, advised Y/N
   1. Name of the victim
   2. Victim ID (enter the patient ID sent automatically) / or Insert text “ID not assigned “if patient has not sought treatment
   3. Village
   4. PEP status: 1) PEP initiated (1^st^ dose received); 2) PEP completed; 3) PEP not sought – advised to seek – URGENT; 4) seeking PEP (1^st^ dose NOT received) – URGENT; 5) PEP not advised.
4. What other animals were bitten by this animal? How many? ☐ None ☐ Dog #___ ☐Cat #___ ☐ Cattle # ___ ☐ Other ( please specify)#________
5. a. Was the animal found? ☐ Yes, ☐ No

b. what was the animal outcome: ☐ alive, ☐ dead, ☐ disappeared **(Tick)**

c. If dead, cause of death: ☐ killed by owner, ☐ by community, ☐ car, ☐ natural causes, ☐ unknown

☐ Killed by animals **(Tick)**

1. What is the animal’s age? (Tick)

☐ Puppy ☐ Puppy (< 3months) ☐ juvenile (<1y) ☐ Adult ☐Unknown

1. What is the animal’s sex? ☐ Male ☐ Female ☐ Unknown
2. Has the animal been vaccinated for rabies?

☐ Yes, what year: _________ ☐ Not vaccinated ☐ Unknown ☐ Not applicable

1. **Risk assessment**
   1. **The Dog/Animal**
      1. **animal signs** (Tick):

- Unprovoked aggression (incl. attempting to bite and grip people, animals, or objects, without feeding
- Excessive salivation
- Unexplained dullness/lethargy
- Hyper sexuality
- Paralysis
- Abnormal vocalization
- Restlessness
- Running without reason
- Tameness/loss of fear of humans (wildlife)
- Active during day (wildlife)

**None of the above**

- 1. Feeding puppies (Yes /No), Eating (Yes /No), Normal behavior i.e. aggressive dog (Yes /No)
  2. **The victim:** Noise (speaking/shouting) (Yes/No); running (Yes/No); Aggressive (Yes/No); scared of dogs (Yes/No); throw anything at the dog (Yes/No); playing (Yes/No); approaching the dog (Yes/No); NO provocation (Yes/No)
  3. **Environment:** Chained (Yes/No); Fenced (Yes/No); with no owner (Yes/No); with owner (Yes/No); Lots of people (Yes/No), Lots of dogs (Yes/No); on its property (Yes/No), Dog came out of nowhere: Tick

1. Rabies Assessment decision: ☐ Healthy ☐ suspicious for of rabies ☐ Sick, not rabies ☐ Unknown (Tick)

1. Was a sample collected? ☐ Yes, date:____________ ☐ No , see 16.1,
   1. If Yes, Location where the sample is stored? ☐ DVO’s office, ☐ house location of LFO, ☐ Vet investigation centre ☐ Other (Specify)
   2. No: Animal disappeared ☐ Decomposed ☐ Body thrown ☐ Burned ☐ Consumed ☐ hyenas ate ☐ Not applicable
2. Lateral flow test done? Yes/ No: Select
3. Test Results: ☐ Positive ☐ Negative ☐ Inconclusive ☐ Unsatisfactory for testing
4. Specimen sent to lab, date: ______________ select
5. **Comments if any: _______________**
